# A mixed-methods evaluation of a large-scale online gatekeeper training to prevent youth suicides in the Netherlands

**DOI:** 10.1186/s12887-026-07005-z

**Published:** 2026-05-21

**Authors:** Paula von Spreckelsen, Natasha Waslam, Manon Merkus, Sanne Rasing, Sabine Jaken, Jeroen Steenmeijer, Renske Gilissen, Saskia Mérelle

**Affiliations:** 1 Research Department, 113 Suicide Prevention, Amsterdam, The Netherlands; 2 GGZ Oost Brabant, Boekel, The Netherlands; 3 Behavioural Science Institute, Radboud University Nijmegen, Nijmegen, The Netherlands; 4 Freelance/independent educational consultant, Amsterdam, The Netherlands; 5 Reinier van Arkel, Den Bosch, The Netherlands; 6 Department of Clinical Psychology, Institute of Psychology, Leiden University, Leiden, The Netherlands; 7 Psychiatry, Amsterdam UMC, Amsterdam, The Netherlands

**Keywords:** Youth suicide, Adolescent suicide, Gatekeeper training, Gatekeeper behavior, Suicide prevention

## Abstract

**Background:**

The increasing trend in suicides among young people emphasizes the urgent need for effective prevention strategies. Gatekeeper trainings (GKTs) equip individuals in a young person’s network with skills to recognize signs of suicidal thoughts and behaviors, initiate supportive conversations, and facilitate referrals to appropriate care. This study examines the reach of a large-scale Dutch online-GKT in training both informal and formal gatekeepers.

**Methods:**

Longitudinal data were collected from Dutch adults who participated in the online-GKT between October 2022 and March 2023. Primary outcomes were self-reported knowledge and self-efficacy before and after the training. Secondary outcomes were training retention and behavior at 3-months follow-up. Repeated measures ANOVAs tested the effects of time, gatekeeper group, and interaction effects while logistic regression analyses explored predictors of gatekeeper behavior. Qualitative analysis examined emerging themes in narrative accounts of gatekeeper behavior.

**Results:**

In total, *n* = 8040 formal gatekeepers (e.g., healthcare workers, educational staff) and *n* = 1142 informal gatekeepers (e.g., family members, friends) participated. Gatekeepers showed significant improvements in self-reported knowledge (*F*(1,7555) = 8761.88, *p* < .001, *η*^*2*^ = 0.54) and self-efficacy (*F*(1,7538) = 4376.75, *p* < .001, *η*^*2*^ = 0.37) directly after the online-GKT. These improvements were maintained over time in the follow-up group (*F*(1.9, 1625.1) = 630.84, *p* < .001, *η*^*2*^ = 0.42; *F*(1.9, 1632.3) = 270.07, *p* < .001, *η*^*2*^ = 0.24). Having engaged in further (in-person) training predicted gatekeepers talking to (*OR* = 1.79[1.30–2.45]) as well as referring (*OR* = 1.56[1.09–2.21]) at-risk adolescents between post-training and follow-up. Qualitative accounts of gatekeeper’s behavior at follow-up revealed benefits (e.g., greater ease in talking to a young person about suicide; acknowledging a young person’s difficulties) alongside challenges (e.g., resistance from young people, limited availability of help).

**Conclusions:**

These findings confirm the importance of online-GKTs in adolescent suicide prevention by reaching a large number of primarily formal gatekeepers and enhancing their self-reported knowledge and self-efficacy. Expanding online-GKTs reach to informal gatekeepers, particularly family members, is crucial given their pivotal role in youth suicide prevention. In addition, improving online-GKTs content to foster interpersonal bonds and promote accessible support resources (e.g., e-health, peer support) may further enhance gatekeeper responsiveness and intervention outcomes.

**Supplementary Information:**

The online version contains supplementary material available at 10.1186/s12887-026-07005-z.

## Background

Suicide is one of the three leading mortality causes among young individuals worldwide [1]. In the Netherlands, it is the most common cause of death in people between the ages of 10 and 30 years old [2]. In the past two decades, suicides among Dutch young adults have increased from 7.3 per 100.000 in 2000 to 10.0 per 100.000 in 2024 [3]. This increasing trend coincides with the decline in youth mental health worldwide [4] and emphasizes the need for effective suicide prevention among young people in the Netherlands.

Many people hesitate to disclose their suicidal thoughts and behaviors. Approximately one half of (young) Dutch people who experience suicidal thoughts do not talk about these thoughts [5, 6]. Communication difficulties are a common factor in youth suicidal behavior [7]. Young people may struggle to express their feelings due to limited vocabulary, poor relationships or mistrust, or unsympathetic reactions when attempting to open up. Hiding these feelings has been linked to (emotional) isolation, particularly the sense of not being understood, which worsens suicidal thoughts and behavior. Shame and fear of stigma, misunderstanding, or unwanted treatment can deter disclosure of suicidal thoughts [8]. For young people, concerns about confidentiality are a major barrier to talking about suicide [9]. Dutch youth emphasize the need for connection, reduced stigma, and the ability to talk openly about their suicidal thoughts and behaviors without fears of repercussions (e.g. [10, 11]). Social support and connection, particularly from informal sources (family, friends), are key to recovery from suicidal thoughts and behavior in young people [12]. Also when it comes to seeking professional help, trust and confidentiality are critical [13]. Factors like relational fractures (e.g., lack of collaboration, broken trust), experiences of stigma, or a lack of control over the treatment can impede young people from forming a bond with a therapist [14]. Building trust is thus essential for young people to feel safe discussing their suicidal thoughts. In sum, research highlights the necessity and importance of creating opportunities for young people to have non-judgmental and empathetic conversations about suicide.

An important and effective role in suicide prevention is played by gatekeepers [15, 16]. Gatekeepers are people in contact with individuals that are at risk for suicide, and thus can act as community safety net for those individuals. Gatekeepers of young people can be individuals in a person’s private network (*informal* gatekeepers; e.g., parents, friends) and professional network (*formal* gatekeepers; e.g., educational staff, health care staff, or sports coaches). Gatekeepers can be trained to identify and respond to individuals at risk for suicide by means of participating in *gatekeeper training* (GKT), which is based on the widely used Question, Persuade, Refer (QPR) method [17]. Systematic reviews indicate that GKTs for gatekeepers of young people are effective in improving gatekeepers’ knowledge and attitudes about suicide, as well as self-efficacy, skills, and the likelihood of talking to and referring young people to mental health treatment [18, 19]. While some findings indicate that GKTs have a positive effect on actual gatekeeper behavior and on suicidal thoughts, behaviors, and referral rates of young people, other research did not find support for these behavioral outcomes [18–24]. Despite mixed findings regarding behavioral outcomes, GKTs appear to be a useful tool for youth suicide prevention by creating opportunities for young people to disclose their suicidal thoughts and behaviors and possibly be referred to appropriate care.

GKTs can be offered in an in-person or online format. Online training has the benefit of being easily accessible to a large target group, being flexible in its use, and being low in costs (e.g. [25, 26]), . Research suggests that online-GKTs are feasible and effective in increasing knowledge, self-confidence/-efficacy, and behavioral intentions in gatekeepers of both adults and adolescents [27–30]. One recent study found that an online GKT was associated with similar improvements in gatekeeper knowledge, preparedness, self-efficacy, and reluctance to intervene as an in-person GKT [31]. In 2021, the Dutch foundation 113 Zelfmoordpreventie has launched an online-GKT training ‘Just-Ask’ (translated from the Dutch title: ‘VraagMaar’) for gatekeepers of adolescents and young adults of approximately 12–25 years of age. This online training is freely accessible and takes approximately 1 h to complete. Due to its high accessibility, the 'Just-Ask' online-GKT aims to reach and train several gatekeeper groups, both in formal settings (e.g., healthcare professionals, educational staff) and informal settings (e.g., family members, peers). The primary aim of the current study is to examine the reach of this large-scale online-GKT in training various formal and informal gatekeeper groups. Specifically, this study intends to [1] identify which types of formal and informal gatekeepers participate in the online-GKT, and [2] assess immediate pre-post training changes in self-reported knowledge and self-efficacy.

The second aim is to explore training retention and gatekeepers’ behaviors at follow-up using a mixed-method approach. Previous research on other GKTs in the Netherlands has found favorable effects of both online and in-person GKTs on gatekeepers’ knowledge and self-confidence, but not on behavioral outcomes [27, 32]. The current study intends to address this critical gap in the literature [18, 19]. As a first step, we examine the retention of self-reported knowledge and self-efficacy in formal and informal gatekeepers at follow-up. As a second step, we explore which factors (including gatekeeper group, (changes in) post training self-reported knowledge and self-efficacy, and engagement in additional training) are associated with a higher likelihood of having engaged in gatekeeper behavior after the training. As a third step, we explore the benefits and challenges that gatekeepers experience when talking to and seeking help with at-risk adolescents, based on their narrative accounts. Together, these findings aim to provide practical recommendations for online-GKTs and for the application of acquired skills for suicide-related conversations with young people in both formal and informal gatekeeper groups.

## Method

### Design

The current study had an observational study design with assessments at three timepoints: pre-training (T1), post-training (T2) and 3-month follow-up (T3).

### Participants

The population of the current study was individuals who (might) encounter adolescents and young adults of approximately 12–25 years old and at risk for suicide. Participants were adults (18 years or older) who signed up to participate in the 'Just-Ask' online-GKT. Participants were included if they started the 'Just-Ask' online-GKT in the period between 1 October 2022 and 11 March 2024 and consented to participate in the scientific evaluation at the start of the training. The Medical Ethical Committee of Amsterdam UMC issued a non-WMO declaration (reference number 2022.0327).

### Materials

#### Intervention

The 'Just-Ask' training for gatekeepers consisted of 3 short lessons, designed to be completed within one hour. According to the QPR GKT method [17], gatekeepers were first taught how to identify signals of and how to ask about suicidal thoughts and behavior (Question). They then learned how to promote the acceptance of help (Persuade) and, finally, how to refer a person to appropriate resources for help and support (Refer). In the first lesson, participants learned how to recognize signals of suicidal thoughts and behaviors in young people. The second lesson consisted of teaching gatekeepers how to ask whether a young person is thinking about suicide and how to discuss suicidal thoughts. In the third lesson, gatekeepers learned about different possibilities for finding help (e.g., involving others; access to professional help; how to deal with challenges such as long waiting lists). Each lesson had practical information in the form of text, animations, videos and interactive formats designed to enhance knowledge and attitudes about youth suicidal thoughts and behaviors and conversation skills. At the end of the training, participants could opt to complete a 10-item multiple-choice knowledge test to receive a certificate. If a minimum of 8 questions were answered correctly, the participants were shown their final score and explanations for any incorrect answers. Those scoring fewer than 8 correct answers were offered to retake the test. In the end, participants were asked to rate the training and to indicate if they would recommend the training. The training was developed by 113 with input from experts, including researchers, clinicians, and people with lived experience. More information on the content of the training (in Dutch) can be found Additional file 1 (see Additional file 1.docx).

#### Instruments

##### Gatekeeper groups

Participants were asked to indicate their motivation for participating in the GKT. First, they selected whether they were taking the training for (a) their work/education or (b) a personal situation. Participants were then asked to identify their role: (a) a health care professional, (b) a teacher or another role in education, (c) a participant completing the training as part of their education, (d) a parent/family member of a young person with (possible) suicidal thoughts, (e) a friend of a young person, (f) a trainer, coach, volunteer of a young person, or (g) other (with an open text field).

##### Self-reported knowledge

Gatekeeper knowledge about suicide was assessed with four items from previous studies [32, 33]: (a) knowledge about suicide in young people, (b) knowledge about signals that point to suicidal thoughts in young people, (c) knowledge of how to talk to young people about suicidal thoughts, and (d) knowledge of how to find help for young people with suicidal thoughts. The items were answered on a 5-point Likert scale ranging from 1 (very little) to 5 (very much). Item scores were summed to calculate total knowledge scores, ranging from 4 (minimum) to 20 (maximum). The internal consistency of the knowledge assessment was good (Crohnbach’s *α* = 0.86). The items (in Dutch) can be found in supplementary file 1.

##### Self-reported self-efficacy

Gatekeeper self-efficacy was assessed with four items from previous studies [32, 33]: three items about confidence in (1) recognizing whether a young person is thinking about suicide, (2) talking to a young person about suicide, and (3) helping a young person with suicidal thoughts, and a fourth item that measured hesitation to ask whether a young person is thinking about suicide. Items were answered on a 5-point Likert scale ranging from 1 (not at all) to 5 (totally). After reverse-coding of the fourth item, scores were summed to calculate total self-efficacy scores, ranging from 4 (minimum) to 20 (maximum). The internal consistency was acceptable (Crohnbach’s *α* = 0.72). The items (in Dutch) can be found in Supplementary File 1.

##### Self-reported gatekeeper behavior

Gatekeeper behavior was assessed at T3 using both closed and open-ended questions.

Quantitative assessment of gatekeeper behavior: Engagement in gatekeeper behavior was assessed using two closed questions: 1. ‘Have you talked to a young person who has suicidal thoughts in the past three months?’ (‘yes’/‘no, I have seen a young person with suicidal thoughts but not talked to them’/‘no, I have not seen a young person with suicidal thoughts’), and 2. ‘Have you helped a young person with suicidal thoughts to find help in the past three months?’ (‘yes’/‘no, I have seen a young person with suicidal thoughts but not helped them to find help’/‘no, I have not seen a young person with suicidal thoughts’). Two binary variables were computed for each behavior, with 1=’yes’, and 0 = all ‘no’ responses (i.e., both types of ‘no’ were merged into a single category).

Qualitative assessment of gatekeeper behavior: After the quantitative questions, the following open-ended questions were asked: ‘What went well in the conversation, and what did you find difficult? (in response to a ‘yes’ on the first closed question); ‘What went well in finding help, and what did you find difficult?’(in response to a ‘yes’ on the second closed question); Would you like to explain why you did not talk to a young person with suicidal thoughts? (in response to a ‘no’ on the first closed question); Would you like to explain why you did not help a young person with suicidal thoughts to find help? (In response to a ‘no’ on the second closed question). These items were created by a panel of experts, including researchers, clinicians, and people with lived experience.

Other training: In addition, the follow-up assessment also included a question on whether gatekeepers engaged in another offline or online suicide prevention training (yes/no).

### Procedure

Adults of the Dutch general population were made aware of the 'Just-Ask' online-GKT via public campaigns (e.g. [34]), and the social media channels of 113. In addition, adults from organizations that work with young people (e.g., schools; healthcare institutions) were invited to participate in the 'Just-Ask' online-GKT by their respective organizations. Participants could access the free 'Just-Ask' online-GKT via the website of 113. Upon clicking on the training, participants were informed about its aims. If they agreed to start, they were asked to create an account with their email-address and to answer the gatekeeper group question. The training did not include a demographic assessment. Participants were then informed that the training consists of three steps, that the training takes approximately 60 min, and that they will receive a certificate upon completion. Before the start of the training, participants were asked whether they were 18 years or older. If they selected ‘yes’, they were asked to provide consent for answering a few questions before and after the training, which will be used for research. Participants who consented were forwarded to the pre-assessment, proceeded with the training, and completed the post-assessment after finishing the training. After three months, participants were contacted via email to participate in the follow-up assessment.

### Analysis

#### Main analysis (reach and short-term changes)

As a first step, gatekeepers were categorized based on their responses to the gatekeeper group question. Because the data on the gatekeeper group question contained many open answers, a Large Language Model [35] was used to classify the data into the following subgroups: professional healthcare, helpline, education, training, other work (e.g. casino, police, church staff, coaches), trainee, student, family, friend, and own interest. The latter three subgroups were categorized as informal gatekeepers, while the former 7 groups were categorized as formal gatekeepers. Second, changes in total knowledge and self-efficacy scores from pre-training (T1) to post-training (T2) were analyzed using repeated measures ANOVAs. Factors included time, gatekeeper group (formal vs. informal), and the interaction between time and gatekeeper group. Significant interaction effects were further examined using pairwise comparisons.

#### Secondary analysis (retention and behavior at follow-up)

The first step of the exploratory analysis was to examine changes in total self-reported self-efficacy and self-reported knowledge scores from pre-training (T1), post-training (T2), to follow-up (T3) using repeated measures ANOVAs. Factors included time, gatekeeper group (formal vs. informal), and the interaction between time and gatekeeper group. Significant interaction effects were further examined using pairwise comparisons with Bonferroni corrections. As a second step, logistic regression analyses were conducted to assess the likelihood of having had a conversation about suicide and engaging in referral behavior. Separate logistic regression models with Holm-Bonferroni corrections were conducted with the following groups of predictors: 1: gatekeeper group (formal/informal), T2 knowledge, T2 self-efficacy, T2 knowledge*T2 self-efficacy; 2: gatekeeper group (formal/informal), T2-T3 knowledge change, T2-T3 self-efficacy change, T2-T3 knowledge change*T2-T3 self-efficacy change; 3: gatekeeper group (formal/informal), further training (yes/no). Point-biserial correlation coefficients with Bonferroni correction were computed to investigate the association between T3 knowledge and T3 self-efficacy with gatekeeper behavior (having had a conversation & engaging in referral behavior). Lastly, qualitative responses to the open-ended questions on gatekeeper behavior were analyzed using a thematic analysis approach (following principals outlined by Braun & Clarke [36]). A combination of deductive and inductive strategies was employed. Given that the questions prompted participants to reflect on what went well and what was challenging, responses were initially categorized deductively into positive aspects and challenges. Subsequently, an inductive coding process was undertaken to identify more nuanced patterns within these categories. Two researchers (PS, NW) independently familiarized themselves with the data, and generated initial codes. They then compared and discussed their coding and interpretations, refining codes and developing themes through an iterative process. Finally, one researcher coded all data using the agreed-upon coding framework with regular consultation between researchers to ensure consistency in coding and interpretation. The quantitative and qualitative results were presented separately but were integrated in the discussion of the findings.

## Results

### Main aim: reach of the online-GKT in training formal and informal gatekeepers

#### Reach

**Fig. 1:**
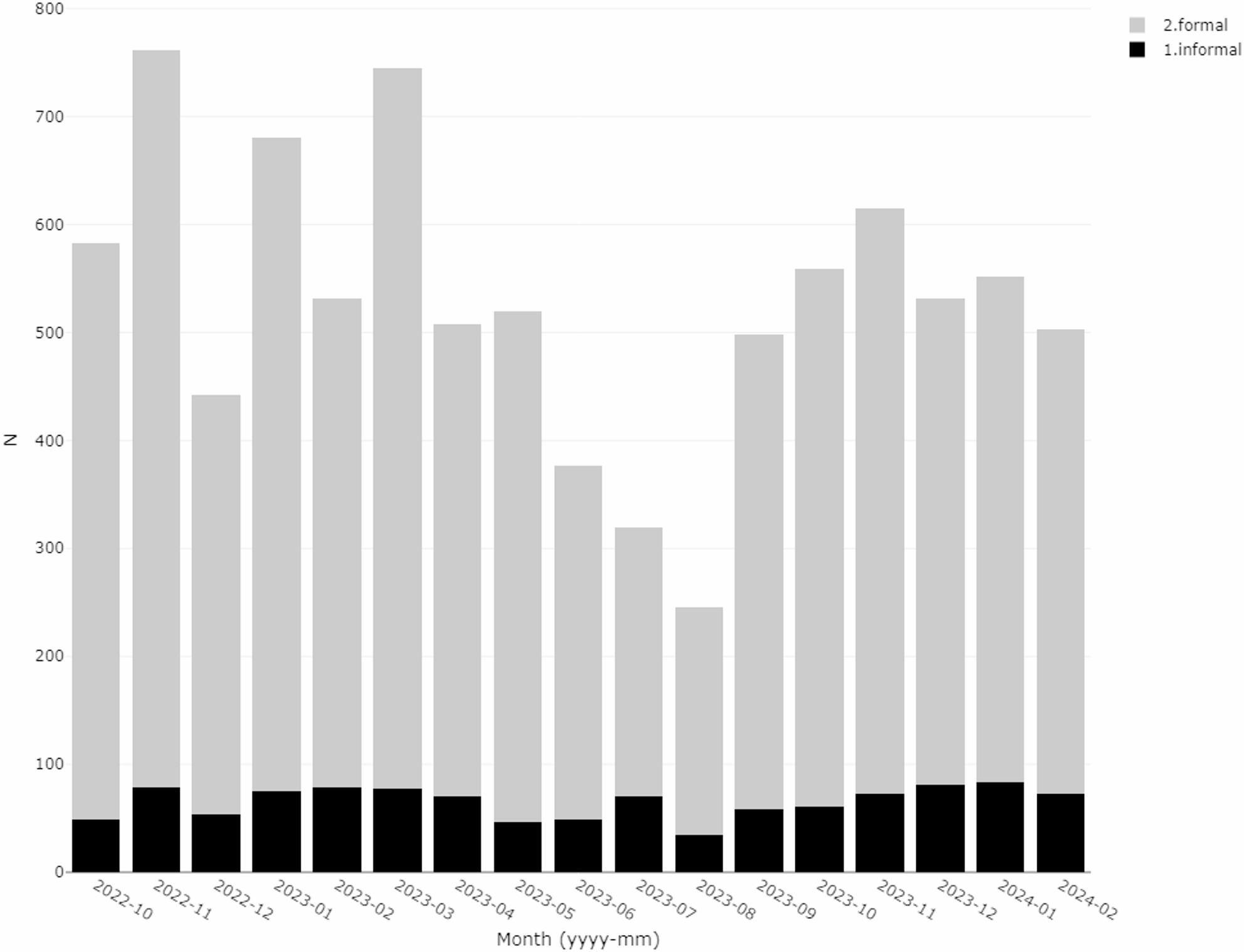
Number of registrations of formal and informal gatekeepers per month

Of the 15347 eligible gatekeepers who participated in the training, *N* = 9182 (59.8%) consented to participate in the study and filled in the pre-training questionnaires. Within the group of formal gatekeepers (*n* = 8040), the majority were professional healthcare workers (47.8%, *n* = 3845), and another 1.7% (*n* = 137) worked at a helpline. In addition, 23.2% (*n* = 1865) of formal gatekeepers worked in educational settings, and 19.8% participated as part of their vocational training (*n*=1502) or study program (*n* = 87). The remaining 7.5% (*n* = 604) consisted of adults in various professions, such as police staff, coaches, casino staff, and church staff/volunteers. Informal gatekeepers (*n* = 1142) were family members (57.7%; *n* = 659) or friends (25.2%; *n* = 288) of young people with suicidal thoughts, as well as individuals who participated for personal reasons or out of interest (17.1%; *n* = 195). Figure 1 presents formal and informal gatekeeper group registrations per month. Of the gatekeepers who filled in the pre-training questionnaires, *N* = 7557 completed the post-training assessment, indicating a dropout rate of 17.7%. Dropout rates differed between gatekeeper groups directly after the online-GKT (14.6% dropout for formal gatekeepers vs. 39.6% for informal gatekeepers). Visual inspection of the distribution of self-efficacy and knowledge scores at pretraining did not reveal differences between participants with complete data and those with incomplete data at post-training. 

#### Immediate training changes in self-reported knowledge and self-efficacy

Table 1 presents the total scores of self-reported knowledge and self-efficacy for formal and informal gatekeepers, in total and per subgroup, at pre-training (T1) and post-training (T2). Table 2 presents the results of the repeated measure ANOVA’s on the immediate changes in self-reported knowledge and self-efficacy.

**Table 1:** Means and standard deviations of self-reported knowledge and self-efficacy scores before training and after training, across and within gatekeeper groups

	Knowledge		Self-efficacy	
	T1	T2	T1	T2
Formal gatekeepers (total)	11.61 (2.76)	16.14 (1.69)	12.29 (2.86)	15.54 (2.20)
Healthcare^1^	12.14 (2.67)	16.21 (1.59)	12.87 (2.72)	15.84 (2.12)
Education	11.09 (2.76)	15.85 (1.70)	11.74 (2.95)	15.17 (2.28)
Training^2^	11.14 (2.67)	16.29 (1.83)	11.62 (2.79)	15.21 (2.16)
Other	11.00 (2.97)	16.19 (1.78)	11.94 (2.92)	15.58 (2.24)
Informal gatekeepers (total)	9.85 (3.32)	15.77 (2.14)	10.73 (2.95)	14.79 (2.60)
Family member	9.06 (3.16)	15.34 (2.10)	10.18 (2.78)	14.37 (2.62)
Friend	10.97 (3.24)	16.42 (2.17)	11.30 (3.06)	15.27 (2.41)
Personal interest	10.88 (3.20)	16.11 (1.98)	11.76 (2.91)	15.31 (2.59)

1 = includes both professional healthcare workers as well as helpline workers; 2 = includes people in vocational training and higher education;

**Table 2:** Results of the repeated measures ANOVA’s on immediate changes in and retention of self-reported knowledge and self-efficacy

	F	df	*p*	η2
Self-reported knowledge				
Immediate changes (T1-T2)				
Time	8761.88	1,7555	< 0.001	0.54
Group	206.95	1,7555	< 0.001	0.03
Time x group	149.37	1,7555	< 0.001	0.02
Retention at follow-up (T1-T2-T3)				
Time*	630.84	1.9, 1625.1	0.003	0.42
Group	8.82	1, 871	< 0.001	0.01
Time x group*	3.12	1.9, 1625.1	0.048	0.00
Self-reported self-efficacy				
Immediate changes (T1-T2)				
Time	4376.75	1, 7538	< 0.001	0.37
Group	166.72	1, 7538	< 0.001	0.02
Time x group	47.15	1, 7538	< 0.001	0.01
Retention at follow-up (T1-T2-T3)				
Time*	270.07	1.9, 1632.3	< 0.001	0.24
Group	11.91	1, 843	< 0.001	0.01
Time x group*	4.19	1.9, 1632.3	0.016	0.01

*Sphericity-corrected

Self-reported knowledge scores significantly increased from pre- to post-training in all gatekeepers, with a large effect size. On average across both time points, formal gatekeepers had significantly higher self-reported knowledge levels than informal gatekeepers. There was a significant interaction effect of time and gatekeeper group. Pairwise comparisons revealed moderately larger self-reported knowledge scores in formal compared to informal gatekeepers at T1 (*t*(1375) = 17.2, *p* < .001 (Bonferroni-corrected), *d* = 0.58), and negligible larger self-reported knowledge scores in formal compared to informal gatekeepers at T2 (*t*(778) = 4.47, *p* < .001 (Bonferroni-corrected), *d* = 0.20). Large increases in knowledge from T1 to T2 were observed in formal gatekeepers (*t*(6866) = 136.0, *p* < .001 (Bonferroni-corrected), *d* = 1.98), as well as in informal gatekeepers (*t*(689) = 49.8, *p* < .001 (Bonferroni-corrected), *d* = 2.12). Both groups scored high on the knowledge test after the training (M_formal_ = 9.55 (SD = 0.70), M_informal_ = 9.44 (SD = 0.94)).

Also, for self-reported self-efficacy, there was a large significant increase over time, meaning that self-efficacy scores increased from pre- to post-training in participants in general. On average across both time points, formal gatekeepers reported significantly higher self-reported self-efficacy scores compared to informal gatekeepers. A significant interaction effect of time and gatekeeper group was observed. Pairwise comparisons revealed moderately larger self-reported self-efficacy scores in formal compared to informal gatekeepers at T1 (*t*(1463) = 16.8, *p* < .001 (Bonferroni-corrected), *d* = 0.54), and slightly larger self-reported self-efficacy scores in formal compared to informal gatekeepers at T2 (*t*(786) = 7.35, *p* < .001 (Bonferroni-corrected), *d* = 0.31). Large increases in self-efficacy from T1 to T2 were observed in formal gatekeepers (*t*(6853) = 98.9, *p* < .001 (Bonferroni-corrected), *d* = 1.27), as well as in informal gatekeepers (*t*(685) = 36.3, *p* < .001 (Bonferroni-corrected), *d* = 1.46).

### Secondary aim: training retention and behavior at follow-up

#### Retention of self-reported knowledge and self-efficacy training changes at follow-up

**Fig. 2:**
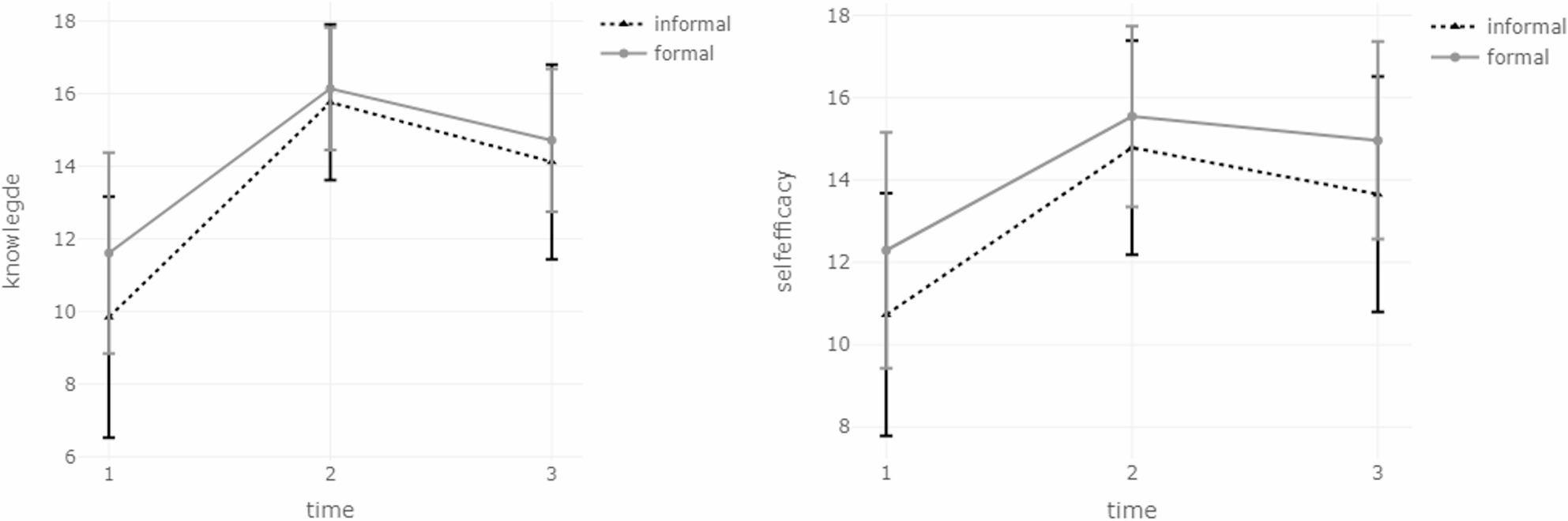
Line graph depicting the means and standard deviations of knowledge and self-efficacy scores before training, after training and at follow-up

At the three-month follow-up, *N* = 913 participants completed the assessment (dropout: 87.9%). Dropout was comparable between gatekeeper groups at follow-up (88.1% dropout formal; 86.4% dropout informal). Visual inspection of the distribution of self-efficacy and knowledge scores at pre- and post-training did not reveal differences between participants with complete data and those with incomplete data at post-training and follow-up. Table 2 presents the results of the repeated measure ANOVA’s on the retention of self-reported knowledge and self-efficacy at follow-up. Figure 2 illustrates mean knowledge and self-efficacy scores at the three measurement occasions (before trainin, after training, follow-up).

In this group of gatekeepers, a repeated measures ANOVA on self-reported knowledge scores across the three measurements revealed significant main effects and interaction effects. Pairwise comparisons revealed slightly larger knowledge scores between formal and informal gatekeepers at follow-up (*t*(99.0) = 2.03, *p* < .005 (Bonferroni-corrected), *d* = 0.25). Large increases in knowledge from T1 to T3 were observed in formal gatekeepers (*t*(785) = 33.9, *p* < .001 (Bonferroni-corrected), *d* = 1.30), as well as in informal gatekeepers (*t*(88) = 10.2, *p* < .001 (Bonferroni-corrected), *d* = 1.42). Moderate decreases in knowledge from T2 to T3 were observed in formal gatekeepers (*t*(783) = -19.3, *p* < .001 (Bonferroni-corrected), *d* = -0.78), as well as in informal gatekeepers (*t*(88) = -6.44, *p* < .001 (Bonferroni-corrected), *d* = -0.68).

A repeated measures ANOVA on self-reported self-efficacy scores across the three measurement occasions revealed significant main effects and interaction effects. Pairwise comparisons revealed slightly larger self-efficacy between formal and informal gatekeepers at follow-up (*t*(96.3) = 4.04, *p* < .001 (Bonferroni-corrected), *d* = 0.50). Large increases in self-efficacy from T1 to T3 were observed in formal gatekeepers (*t*(762) = 24.7, *p* < .001 (Bonferroni-corrected), *d* = 1.01), as well as in informal gatekeepers (*t*(83) = 6.09, *p* < .001 (Bonferroni-corrected), *d* = 1.01). Small decreases in self-efficacy from T2 to T3 were observed in formal gatekeepers (*t*(760) = -9.0, *p* < .001 (Bonferroni-corrected), *d* = -0.25), as well as in informal gatekeepers (*t*(83) = -5.24, *p* < .001 (Bonferroni-corrected), *d* = -0.41) (Fig. 2).

#### Gatekeeper behavior

Of all 913 gatekeepers at the follow-up assessment, *n* = 362 (39.6%) gatekeepers had a conversation about suicide and *n* = 245 (26.8%) supported a young person in seeking help.

#### Determinants of engaging in gatekeeper behavior

The logistic regression analyses on (a) having a conversation about suicide, and (b) supporting a young person in seeking help (referral) revealed that informal gatekeepers were significantly more likely to have had a conversation about suicide with a young person within three months after the training than formal gatekeepers (OR = 0.57[0.37–0.87]). Having engaged in further training in the period between participation in the training and follow-up was significantly predictive of a higher likelihood of having had a conversation about suicide (OR = 1.79 [1.30–2.46]) and supporting a young person in seeking help (OR = 1.56[1.09–2.21]). All other predictors (T2 knowledge, T2 self-efficacy, T2 knowledge*T2 self-efficacy; T2-T3 knowledge change, T2-T3 self-efficacy change, T2-T3 knowledge change*T2-T3 self-efficacy change) were not found to be statistically significant (see Table A1 in Additional File 2 for the full results). Point-biserial correlation coefficients revealed small positive associations between self-reported knowledge and self-efficacy with gatekeeper behavior at follow-up (r’s 0.14–0.18; see Table A2 in Additional File 2 for the full results).

#### Practical experiences of gatekeepers behavior

In total, 239 gatekeepers provided qualitative data on their experience of engaging in gatekeeper behavior. Many of these descriptions stemmed from formal gatekeepers (87%).

##### Confidence

Approximately a third of gatekeepers (36%) reflected on their confidence of engaging in gatekeeper behavior.

Positive aspects: Many reported increased ease or calmness in conversations about suicide with young people (16%). For example, gatekeepers were less afraid or hesitant to ask about suicidal thoughts, felt more comfortable and relaxed, and experienced these conversations as more organic.

*I dare to ask more questions and use concrete words like […] suicide. I no longer get so nervous discussing the subject and am less afraid of doing it ‘wrong’.*

*Challenges:* A few gatekeepers continued to experience conversations about suicide as stressful/tricky and found it difficult to cope with their own emotions.

*[…] It remains difficult to not let it touch you too much emotionally*,* because it is an intense subject and you also care about the student.*

#### Interpersonal connection

Positive aspects: Around 44% of gatekeepers reported positive interpersonal experiences during conversations. Common themes included acknowledging the young person’s experience (16%), meaning that they took the difficulties seriously and showed understanding through validation or reassurance. Gatekeepers also reported on creating a space of openness and honesty, establishing a supportive connection or offering a listening ear.

*An open and honest conversation. Asking a question and expressing concern resulted in an emotionally valuable conversation.*

Challenges: Around one fifth of gatekeepers (21%) reported interpersonal challenges, such as dealing with resistance from young people (e.g., young people not wanting their parents to be informed; 13%). Another challenge was to deal with hopelessness expressed by young people with suicidal thoughts.

*It was difficult to discuss that I was going to notify the parents. This student absolutely did not want that. Then I gave her the choice to choose between parents or a general practitioner. She then chose the general practitioner.*

#### Application of gatekeeper skills

Positive aspects: Most respondents (74%) described successfully applying gatekeeper skills. These included directly asking about suicide (29%) and continuing to ask for more details on suicidal thoughts, plans, or behavior (34%). Respondents also appreciated more knowledge about the severity of suicidal thoughts and behaviors, structuring conversations, and following the training guidelines on creating hope.

*I dare to ask more about how she is really doing and if she is still hurting herself and thinking about death*.

Challenges: Some gatekeepers (13%) described difficulties applying gatekeeper skills (e.g., directly asking about suicidal thoughts, structuring conversations), especially in complex cases involving suicidal behavior or persistent suicidality.

*It went well. Except these are much more intense conversations than came up in the training. We’ve had some students who have actually made a suicide attempt […]*.

#### Supporting young people in finding help

Positive aspects: Approximately two thirds (62%) reported supporting young people in finding help. This included referring the young person to professional help (such as the general practitioner, psychologist, or the 113 helpline; 31%), but also informing parents, making (safety)plans and maintaining contact with the young person. Some gatekeepers collaborated with colleagues.

*She already has help*,* but we have been looking mainly at whether this is enough or whether we need to implement something else. We will have a consultation on that soon with her and anyone around her that she wants involved.*

Challenges: Around 30% of gatekeepers reported challenges, often related to the availability or adequate help (e.g., long waiting lists for psychological treatment; 8%). Other than that, gatekeepers struggled with assessing treatment needs, making follow-up agreements, coping with feelings of responsibility, and involving a young person’s (dysfunctional) environment.

*It is tricky to judge what kind of help is needed. You want to get it right. […] Referral is possible but is also difficult with young people with suicidal thoughts […]. There are waiting times*,* or clients with suicidal behavior are not admitted by some institutions.*

#### Effect of gatekeeper behavior on the young person

Around a third of gatekeepers (32%) described the effects of their behavior on young people. Gatekeepers described that the young person found help (e.g., at the general practitioner, psychologist, or the 113 helpline; 12%). Next to that, gatekeepers also described that conversations resulted in increased openness, contentment, relief, or wellbeing in young people.

*I listened to the person without judgment. Together with the person*,* we told it to the counselors*,* so now he has the help that he needs.*

#### Reasons for not engaging in gatekeeper behavior

In total, 63 gatekeepers (of which 54 formal gatekeepers) provided reasons for not engaging in gatekeeper behavior. Common themes were the presence of existing support, intervention by another person, or the young person’s lack of need to talk or seek help.

## Discussion

### Summary of findings

This study evaluated a brief online gatekeeper training (GKT) for gatekeepers of young people. Regarding the main aim of the study, various groups of formal and informal gatekeepers participated in the online-GKT. Among the 8040 formal and 1142 informal gatekeepers we found large improvements in self-reported knowledge and self-efficacy from before to after training. Exploratory analysis indicated retention of improvements in self-reported knowledge and self-efficacy at follow-up. Participation in additional training was the only predictor that was significantly associated with a higher likelihood of engaging in both gatekeeper behaviors (talking to and referring at-risk adolescents). Narrative accounts of gatekeepers’ behavior at follow-up revealed benefits (e.g., greater ease in talking to a young person about suicide; acknowledging a young person’s difficulties) alongside challenges (e.g., resistance from young people, limited availability of help).

### Reach of the online-GKT in training formal and informal gatekeepers

The online-GKT predominantly reached formal gatekeepers, especially those in healthcare (e.g., clinical psychologists, general practitioners, nurses) or educational settings (teachers, school directors, student counsellors). Healthcare and educational professionals represent important nodes within the gatekeeper safety net for young people. For instance, 60% of Dutch young people who died by suicide had been in treatment at a specialized mental health care facility [37], and the number of Dutch young people seeing a general practitioner because of suicidal thoughts is rising [38]. Healthcare staff thus frequently encounter young people at risk of suicide. The same applies to the educational setting, which can serve as a protective factor in youth suicide prevention [39]. Additionally, the diversity of professional backgrounds of respondents (e.g., police staff, coaches, casino staff, and church staff/volunteers) suggests a broad applicability of the training. However, informal gatekeepers (e.g. family members and friends of young people) were underrepresented, despite the recognized importance of family and peers to youth suicide prevention [40, 41]. Expanding outreach to informal gatekeepers therefore appears critical. The observed increases in self-reported knowledge and self-efficacy suggest that this low-cost training tool is effective in educating diverse population groups and may help reduce taboos. This applies to both formally trained gatekeepers with some pre-existing knowledge and particularly to informal gatekeepers with less prior knowledge and self-efficacy. Thus, the online-GKT aligns with the needs of young Dutch people for more openness, knowledge and less taboo about suicide [10]. The observed improvements in self-reported knowledge and self-efficacy are comparable to those of in-person GKT in the Netherlands [32].

### Training retention and behavior at follow-up

Secondary analyses in gatekeepers that participated in the follow-up assessment indicated that improvements in self-reported knowledge and self-efficacy were maintained over time. This replicates earlier findings of online GKTs [27]. However, only a small percentage (10%) provided follow-up data, limiting the generalizability of the results due to selection bias. Still, the findings point to the potential of online-GKTs being associated with maintained changes in self-reported knowledge and self-efficacy. At follow-up, nearly half of the respondents had talked to a young person, and one fourth had referred a young person to help. Neither post-training knowledge or self-efficacy nor changes in knowledge or self-efficacy from post-training to follow-up were found to be associated with a higher likelihood of any behavioral outcome. Immediately increased knowledge and confidence from online training may thus not fully translate to practice. Participation in additional suicide prevention training was associated with engagement in both gatekeeper behaviors, suggesting that synchronous in-depth training may be necessary for gatekeepers to feel more comfortable applying skills in real-life situations. Finally, informal gatekeepers were more likely to talk to young people compared to formal gatekeepers. Differences in sample sizes and selection bias might explain these findings as informal gatekeepers may participate to talk to a specific young person in their environment, while formal gatekeepers participate for general training purposes.

#### Positive aspects of gatekeeper behavior

The reported positive experiences in applying conversational skills (e.g., directly asking about suicidal thoughts and behaviors) and making referrals indicate that some gatekeepers could successfully apply the training in practice. While self-reported self-efficacy immediately after the training was not a predictor of engagement in gatekeeper behavior, the narrative accounts suggest that the training could facilitate confidence in some gatekeepers. Higher self-efficacy scores at follow-up were associated with a higher likelihood of having engaged in gatekeeper behavior. This might imply that (positive) learning experiences are necessary to build confidence in discussing suicide with young people. Nearly half of the respondents described positive interpersonal aspects, such as acknowledging difficulties, active listening, and establishing a supportive connection. As noted earlier, many people do not disclose their suicidal thoughts [5] and may experience feelings of shame, self-stigma, and fear related to disclosing their suicidal thoughts and behaviors [8, 42]. Disclosure is more likely when individuals perceive the listener as trustworthy, supportive, empathic and validating [43]. Especially for young people, the quality of the relationship with healthcare providers has been identified as a crucial factor for treatment outcomes [12, 44, 45]. Therefore, supportive interpersonal behavior by gatekeepers appears critical in encouraging disclosure. In addition to reports of general positive outcomes on young people (e.g., referrals to care, relief), these practical experiences indicate that online-GKT may be a valuable tool for promoting multiple facets of gatekeeper behavior.

#### Challenges of gatekeeper behavior

Some gatekeepers reported difficulties in applying gatekeeper skills, such as directly asking about suicide or structuring conversations. This suggests that the online-GKT may not fully support all participants in developing sufficient confidence and comfort. Another identified key challenge was dealing with young people’s resistance to opening up or involving parents. As previously noted, family members play an important role in youth suicide prevention [12, 40, 41]. Yet, young people often experience feelings of shame and burdensomeness, and fear repercussions/loss of autonomy (e.g., involuntary hospitalization) when it comes to disclosing their suicidal thoughts to family [46, 47]. The quantitative results of the current study indicate that informal gatekeepers, such as family members, benefit from online-GKT just as much as formal gatekeepers. Providing online-GKT training to family members may therefore represent a promising suicide prevention strategy for increasing openness about suicidal thoughts and behavior in the family context and addressing this barrier. At the same time, some gatekeepers experienced difficulties involving parents in situations where the family environment was dysfunctional. Family dysfunction and problems, such as low parental care or parental neglect, are risk factors for suicidal thoughts and behaviors [48–54]. Applying gatekeeper skills was challenging in complex cases, such as those involving persistent suicidality. These cases are often characterized by a greater emotional dysregulation, hopelessness, more self-harm, the development of suicidal identity, and an elevated risk for suicide [55–58]. Such complexity may require more advanced skills and support than can be provided through brief online training alone.

### Limitations

The observational design of this study does not allow us to draw conclusions about causality. Moreover, because behavior was only assessed at follow-up, we cannot make any conclusions on changes in gatekeeper behavior associated with the online-GKT. There was a very high (almost 90%) participant attrition rate at follow up, which is comparable to similar GKT studies [30, 31]. Although analysis of non-responders revealed no differences, we cannot exclude a selection bias (e.g., highly motivated next-of-kin or individuals ‘bound’ by organizational obligations might have been more likely to complete the follow-up assessment). Due to the differences in group sizes between formal and informal gatekeepers, the follow-up (qualitative) data is mainly representative of formal gatekeepers. To be able to generalize the current follow-up findings to gatekeepers in general, replication studies in large samples with less dropout, and investigations of drop-out reasons, are imperative. It should also be noted that the gatekeeper groups are not mutually exclusive (e.g., a teacher may not only act as gatekeeper in their professional environment but also in their private life as a parent). Critically, all assessments relied solely on self-report, and we have no data on the outcomes of gatekeeper conversations for young people such as treatment referral rates. Thus, the reported changes reflect the gatekeepers’ perceptions rather than objective changes. Finally, qualitative data were collected via online forms rather than in-depth interviews, limiting the depth of responses (e.g., on barriers to engaging in gatekeeper behavior).

### Recommendations

Based on the current results, we recommend implementing strategies to increase the reach of online-GKTs to informal gatekeepers, particularly family members of at-risk youth. A key next step is to examine which strategies are effective to engage informal gatekeepers and encourage their participation in suicide prevention training. Possible strategies could include targeted campaigns (e.g. [59]), or accessible online micro-learnings. Since informal gatekeepers play a vital role in fostering openness around suicide, expanding their participation may increase young people’s willingness to disclose. To mitigate declines in self-efficacy and knowledge, and address high attrition, we recommend automatic reminders and refresher content post-training to reinforce learning points.

To further promote gatekeeper behavior, online-GKT’s should encourage participation in additional synchronous in-depth training, either in-person or online, that touches on the identified challenges (e.g., dealing with resistance, applying gatekeeper skills in complex cases; [60]). This is particularly important because equipping gatekeepers with advanced skills for more complex contexts (e.g., young people with persistent suicidality; dysfunctional family environments) goes beyond the capacity of short online-GKTs. Online-GKT’s can however serve as a gateway to more advanced training, supportive resources (e.g. [61, 62]), or clinical guidelines [63, 64], helping gatekeepers navigating complexity. To address barriers of low availability of professional help, online-GKTs could link to informal sources of support, such as e-health programs [65] or peer support groups [66, 67].

As of now, the literature on the effect of GKT’s on gatekeeper behavior and on young people has been inconclusive [18–24]. Although the current results indicate potentially promising changes in gatekeeper behavior and young person’s behavior (e.g., treatment referrals, increased openness), more research is necessary to substantiate such possible effects. Specifically, large RCTs should assess (long-term) outcome assessments on gatekeeper behavior as well as on actual referral rates, suicidal behavior, and perceived openness to talk about suicide in young people. Finally, research should explore factors discouraging gatekeepers from engaging in gatekeeper behavior.

## Conclusion

The current findings confirm the importance of online-GKTs in youth suicide prevention by reaching a large number of gatekeepers and enhancing gatekeepers’ self-reported knowledge and self-efficacy. Expanding the reach to informal gatekeepers, particularly family members, is crucial given their pivotal role in youth suicide prevention. In addition, nudging gatekeepers to further training, targeted resources, and accessible care options (e.g., e-health, peer support) may further enhance gatekeeper behavior and outcomes.

## Supplementary Information

Additional file 1.

Aditional file 2.

## Data Availability

The datasets used and/or analyzed during the current study are available from the corresponding author on reasonable request.

## Abbreviations

GKT: Gatekeeper training
