# Supplementary material for: A mixed-methods evaluation of a large-scale online gatekeeper training to prevent youth suicides in the Netherlands
Source: BMC Pediatr. 2026 May 21;26:657. doi: 10.1186/s12887-026-07005-z (PMC13371545; doi:10.1186/s12887-026-07005-z)
Supplement: Supplementary file 1 — Additional file 1. [file 12887_2026_7005_MOESM1_ESM.docx]

**Additional file 1**

***Opbouw van de online training suïcidepreventie gatekeepers jeugd***

| **Start training** |  | |
| --- | --- | --- |
| **Voormeting** | vragen over leeftijd, soort professional of naaste, zelfgerapporteerde kennis en vertrouwen in vaardigheden. | |
| **Les 1: Signaleer** | **Les 2: Praat over suïcide** | **Les 3: Zoek samen hulp** |
| Impactvideo jongere | Interactieve video gesprek docent met jongere | Impactvideo gatekeeper hulp zoeken |
| Cijfers in perspectief: vragen voorkennis en infographic | Gespreksvaardigheden: keuze reacties docent en feedback | Betrekken anderen en meldplicht |
| Misverstanden: stellingen over zelfdoding | Uitdagingen en handvaten gesprek, inclusief sociale media: stellingen over gesprek | Obstakels hulp zoeken |
| Animatie: hoe onstaan suicidale gedachten | Voor jezelf zorgen als gatekeeper: video | Waar kan iemand heen voor hulp |
| Signalen herkennen: portretten jongeren lezen en stellingen | Samenvatting les, introductie doel volgende les | Samenvatting les |
| Risicofactoren: toelichting lezen |  | Keuze certificaat met toetsing of afronden training zonder certificaat |
| Samenvatting les, introductie doel volgende les |  |  |
| **Certificaat en toetsing** |  | |
| **Online toets** | 10 meerkeuzevragen, certificaat is behaald indien 8 van de 10 vragen, herkansen is mogelijk (zo vaak als deelnemer wil). | |
| **Einde training** |  | |
| **Nameting** | vragen over kennis en vertrouwen in vaardigheden en waardering training en uitnodiging voor follow-up onderzoek | |
| **Follow-up training** |  | |
| **Na 3 maanden** | vragen over deelname extra training, positieve en negatieve ervaringen met een gesprek voeren met jongere(n) met suïcidale gedachten en samen hulp zoeken, zelfgerapporteerde kennis en vaardigheden | |

**Vragen**

***Knowledge***

| Vragen | Onderwerp | Vraag/tekst | Antwoordopties |
| --- | --- | --- | --- |
|  |  | Hoeveel weet je van de volgende onderwerpen? | (deze 4 vragen in 1 scherm met horizontaal radio buttons) |
| V4 | Kennis | Kennis over zelfdoding bij jongeren | - Heel weinig – Weinig – Niet weinig/Niet veel – Veel – Heel veel |
| V5 | Signaleren | Signalen die wijzen op gedachten aan zelfdoding bij jongeren | - Heel weinig – Weinig – Niet weinig/Niet veel – Veel – Heel veel |
| V6 | Gesprek voeren | Hoe ik met een jongere moet praten over gedachten aan zelfdoding | - Heel weinig – Weinig – Niet weinig/Niet veel – Veel – Heel veel |
| V7 | Hulp zoeken | Hoe ik hulp kan vinden voor een jongere die denkt aan zelfdoding | - Heel weinig – Weinig – Niet weinig/Niet veel – Veel – Heel veel |

***Self-efficacy***

| Vragen | Onderwerp | Vraag/tekst | Antwoordopties |
| --- | --- | --- | --- |
|  |  | Stel je voor dat je in contact bent met een jongere die mogelijk denkt aan zelfdoding. Geef aan in hoeverre het volgende in die situatie bij je past: | (deze 4 vragen in 1 scherm met horizontaal radio buttons) |
| V8 | Signaleren | Ik heb vertrouwen dat ik kan signaleren of een jongere aan zelfdoding denkt | - Helemaal niet – Een beetje – Neutraal – Nogal - Helemaal |
| V9 | Gesprek voeren | Ik heb vertrouwen dat ik op de juiste manier met een jongere over zelfdoding kan praten | - Helemaal niet – Een beetje – Neutraal – Nogal - Helemaal |
| V10 | Hulp bieden | Ik heb vertrouwen dat ik een jongere met gedachten aan zelfdoding kan helpen | - Helemaal niet – Een beetje – Neutraal – Nogal - Helemaal |
| V11 | Vraag stellen | Ik aarzel om te vragen of een jongere denkt aan zelfdoding | - Helemaal niet – Een beetje – Neutraal – Nogal - Helemaal |
