# Supplementary material for: A mixed-methods evaluation of a large-scale online gatekeeper training to prevent youth suicides in the Netherlands
Source: BMC Pediatr. 2026 May 21;26:657. doi: 10.1186/s12887-026-07005-z (PMC13371545; doi:10.1186/s12887-026-07005-z)
Supplement: Supplementary file 2 — Aditional file 2. [file 12887_2026_7005_MOESM2_ESM.docx]

**Additional file 2**

**Table A1**

**Logistic regression models on gatekeepers having a conversation and seeking help**

|  |  | | **Yes (%)** | **No (%)** | **OR** | **95% CI** |
| --- | --- | --- | --- | --- | --- | --- |
| ***Having a conversation*** | | | |  |  |  |
|  | GK group | |  |  | 0.57* | 0.37-0.87 |
|  |  | Formal | 313 (38.2) | 506 (61.8) |  |  |
|  |  | Informal | 49 (52.1) | 45 (47.9) |  |  |
|  |  | |  |  |  |  |
|  | *GK group +* | |  |  |  |  |
|  | T2 Knowledge | |  |  | 1.09 | 1.00-1.20 |
|  | T2 Self-efficacy | |  |  | 1.08 | 1.01-1.63 |
|  | T2 Knowledge*Self-efficacy | |  |  | 1.01 | 0.98-1.03 |
|  |  | |  |  |  |  |
|  | *GK group +* | |  |  |  |  |
|  | T2-T3 Knowledge | |  |  | 1.06 | 0.99-1.15 |
|  | T2-T3 Self-efficacy | |  |  | 1.01 | 0.94-1.09 |
|  | T2-T3 Knowledge*Self-efficacy | |  |  | 0.99 | 0.97-1.02 |
|  |  | |  |  |  |  |
|  | *GK group +* | |  |  |  |  |
|  | Further training | |  |  | 1.79* | 1.30-2.46 |
|  |  | Yes | 102 (49.3) | 105 (50.7) |  |  |
|  |  | No | 260 (36.2) | 446 (63.2) |  |  |
|  |  | |  |  |  |  |
| ***Referral*** | | | |  |  |  |
|  | GK group | |  |  | 0.74 | 0.46-1.21 |
|  |  | Formal | 216 (28.5) | 543 (71.5) |  |  |
|  |  | Informal | 29 (34.9) | 54 (65.1) |  |  |
|  |  | |  |  |  |  |
|  | *GK group +* | |  |  |  |  |
|  | T2 Knowledge | |  |  | 1.09 | 1.01-1.18 |
|  | T2 Self-efficacy | |  |  | 1.09 | 0.99-1.21 |
|  | T2 Knowledge*Self-efficacy | |  |  | 1.01 | 0.98-1.04 |
|  |  | |  |  |  |  |
|  | *GK group +* | |  |  |  |  |
|  | T2-T3 Knowledge | |  |  | 1.09 | 1.00-1.18 |
|  | T2-T3 Self-efficacy | |  |  | 1.00 | 0.92-1.08 |
|  | T2-T3 Knowledge*Self-efficacy | |  |  | 1.00 | 0.98-1.02 |
|  |  | |  |  |  |  |
|  | *GK group +* | |  |  |  |  |
|  | Further training | |  |  | 1.56* | 1.09-2.21 |
|  |  | Yes | 66 (35.9) | 118 (64.1) |  |  |
|  |  | No | 179 (27.2) | 479 (72.8) |  |  |

Note. GK group was included as a predictor in all models; *p < .0125 (Holm-Bonferroni adjusted p-value for multiple comparisons)

**Table A2**

**Point-biserial Correlation Coefficients of Self-reported Knowledge and Self-efficacy with Gatekeeper Behavior at Follow-up**

|  | **Having a conversation** | **Referral** |
| --- | --- | --- |
| **T3 Knowledge** | 0.17* | 0.18* |
| **T3 Self-Efficacy** | 0.15* | 0.14* |

*p < .01 (Bonferroni adjusted)
